# Metamaterial-Integrated High-Gain Rectenna for RF Sensing and Energy Harvesting Applications †

**DOI:** 10.3390/s21196580

**Published:** 2021-10-01

**Authors:** Woosol Lee, Suk-il Choi, Hae-in Kim, Sunghyun Hwang, Saeyoung Jeon, Yong-Kyu Yoon

**Affiliations:** Department of Electrical and Computer Engineering, University of Florida, Gainesville, FL 32611, USA; leewoosol@ufl.edu (W.L.); sukilchoi@ufl.edu (S.-i.C.); kimhaein@ufl.edu (H.-i.K.); sunghyun.hwang@ufl.edu (S.H.); s.jeon@ufl.edu (S.J.)

**Keywords:** metamaterial, rectenna, RF energy harvesting, high-gain antenna, RF–DC conversion efficiency, rectifier

## Abstract

This paper presents a metamaterial (MTM)-integrated high-gain rectenna for RF sensing and energy harvesting applications that operates at 2.45 GHz, an industry, science, medicine (ISM) band. The novel MTM superstrate approach with a three-layered integration method is firstly introduced for rectenna applications. The integrated rectenna consists of three layers, where the first layer is an MTM superstrate consisting of four-by-four MTM unit cell arrays, the second layer a patch antenna, and the third layer a rectifier circuit. By integrating the MTM superstrate on top of the patch antenna, the gain of the antenna is enhanced, owing to its beam focusing capability of the MTM superstrate. This induces the increase of the captured RF power at the rectifier input, resulting in high-output DC power and high entire end-to-end efficiency. A parametric analysis is performed in order to optimize the near-zero property of the MTM unit cell. In addition, the effects of the number of MTM unit cells on the performance of the integrated rectenna are studied. A prototype MTM-integrated rectenna, which is designed on an RO5880 substrate, is fabricated and characterized. The measured gain of the MTM-integrated rectenna is 11.87 dB. It shows a gain improvement of 6.12 dB compared to a counterpart patch antenna without an MTM superstrate and a maximum RF–DC conversion efficiency of 78.9% at an input RF power of 9 dBm. This results in the improvement of the RF–DC efficiency from 39.2% to 78.9% and the increase of the output DC power from 0.7 mW to 6.27 mW (a factor of 8.96 improvements). The demonstrated MTM-integrated rectenna has shown outstanding performance compared to other previously reported work. We emphasize that the demonstrated MTM-integrated rectenna has a low design complexity compared with other work, as the MTM superstrate layer is integrated on top of the simple patch antenna and rectifier circuit. In addition, the number of MTM units can be determined depending on applications. It is highly envisioned that the demonstrated MTM-integrated rectenna will provide new possibilities for practical energy harvesting applications with improved antenna gain and efficiency in various IoT environments.

## 1. Introduction

With the development of wireless technologies, the ambient wireless power density is growing owing to the increasing number of electromagnetic (EM) power sources. Harvesting this radio frequency energy has gained a lot of attention in recent years. A rectenna (rectifier + antenna), which is the key component of the wireless RF energy harvesting, continues to be a highly expected solution to the power challenges related to ubiquitous sensor connectivity as envisioned in the Internet of Things (IoT). Rectennas with a high-gain antenna and a high-efficiency rectifier are highly required.

Recently, various rectenna designs have been proposed in the literature for RF energy harvesting, such as monopole rectennas [1,2], dipoles [3,4,5], circularly polarized (CP) antennas [6,7,8], dual-band rectennas [9,10], and a broadband rectenna [11]. In order to solve the limited power challenges, researchers have studied various techniques for improving the gain of the rectenna, such as reflecting surface [4,12], differential microstrip [13], differential feeding [14], antenna array [15,16], SIW cavity [17], air gap [9], and aperture coupling [9,18]. In [12], a 5.8 GHz high-gain dual-rhombic-loop rectenna was investigated to achieve an antenna gain of 10.7 dBi. By having a dual-rhombic-loop antenna structure with terminated gaps, left-hand circular polarization was achieved. The maximum RF–DC conversion efficiency was 80% at an input power of 20 dBm. This dual-rhombic-loop structure had an advantage of high gain and broadband properties, but the improved gain was still limited, and an integrated rectifier made the entire system large. In [13], a differential microstrip antenna with a gain of 8.5 dBi was introduced. The differential antenna was utilized in either center-grounded or differential configuration. The rectenna was designed to operate at the GSM900 band (890–960 MHz), with a peak RF–DC conversion efficiency of 65% at an input power of 2.19 dBm. However, a two-layered differential patch antenna with the additional ground layer made the design complexity very high. In [16], a monopole wideband fractal antenna array fed by a microstrip-to-slot-line transition feeding network was designed. As the feeding line was based on slot-line stubs and radial microstrip, the broadband microstrip-to-slot-line transitions were realized. An antenna gain of 8.5 dBi and RF–DC conversion efficiency of 76% at 5 dBm were obtained. This fractal antenna array had an advantage of broadband property (0.91 to 2.55 GHz), but the design process of the fractal antenna array increased the design complexity. In addition, the rectifier was connected to the antenna in a perpendicular direction, thereby increasing the overall dimension of the rectenna and limiting its practical applicability. In [17], a 24 GHz substrate integrated waveguide (SIW) cavity-backed antenna array was demonstrated for millimeter-Wave energy harvesting. By having the SIW cavity in the antenna, the gain was increased from 10.3 dBi to 12.6 dBi. The maximum RF–DC conversion efficiency was 42% at 12.55 dBm. Even though the gain of the demonstrated antenna was relatively high, the measured RF–DC conversion efficiency was too low, and the overall normalized device was comparably large (0.776 λ^3^). Moreover, the SIW cavity made the fabrication and design process more difficult. In [9], a microstrip patch antenna with a right-angle triangular aperture slot was exhibited. By having an air gap between the patch antenna and feed line, the improved antenna gain was realized. An antenna gain of 7.82 dBi and an RF–DC conversion efficiency of 32.5% at 10 dBm were obtained, but the rectifier circuit was not integrated to the antenna, thereby increasing overall system size, as the rectifier was connected to the same layer of antenna. Overall, the improved antenna gains of the previously reported work were still limited, and the methods to enhance the gain highly increased the device complexity. Therefore, a fully integrated rectenna with high gain, low design complexity, and high RF–DC conversion efficiency is needed.

**Figure 1:**
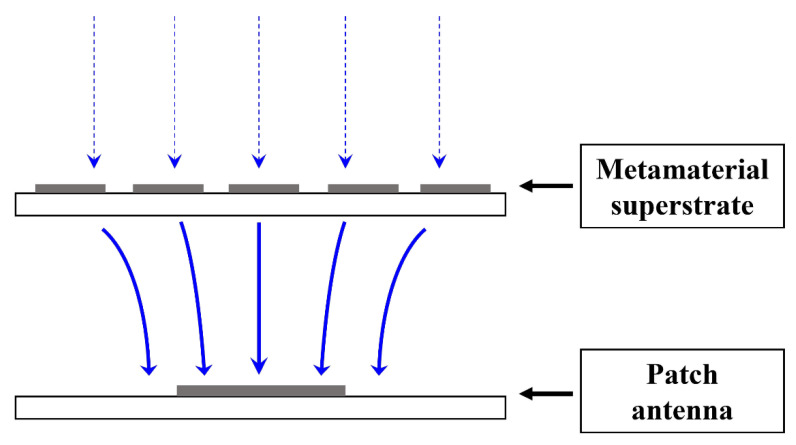
Principle of a gain improvement property of the MTM superstrate.

In the meantime, some researchers have introduced metamaterials (MTMs) for the antenna gain improvement as a form of MTM superstrates [19,20,21,22], where MTM superstrates help increase the antenna gain by utilizing the near-zero refraction characteristic of the MTM. MTMs are artificially designed materials that have uncommon EM properties, such as evanescent wave amplification, negative refraction, and near-zero refraction, thereby improving the antenna gain [23,24]. Figure 1 shows the principle of a gain improvement property of an MTM superstrate. The MTM superstrate focuses and amplifies the EM field at the boundary, owing to its near-zero refractive index, improving the gain of the antenna. By simply employing the MTM superstrate layer on top of the antenna, the gain improvement of the patch antenna can be achieved.

To the best of our knowledge, these novel properties of the MTM superstrate have not been fully investigated in the field of RF energy harvesting. In this work, the novel MTM superstrate approach with a three-layered integration method is firstly introduced for rectenna applications. The MTM-integrated high-gain rectenna, which consists of a first layer of MTM superstrate, second layer of a patch antenna, and third layer of rectifier circuit, is demonstrated. The integrated MTM superstrate enhances the gain of the antenna, inducing the increase of the captured RF power at the rectifier input. It will lead to high-output DC power and high entire efficiency. In addition, the rectifier circuit is incorporated with the backside of the patch, which is isolated from the patch antenna ground plane. While the cross-coupling between the rectifier and the MTM superstrate is suppressed, the rectenna system footprint remains the same. The demonstrated MTM-integrated rectenna solves the power challenges of the rectenna systems owing to its high-gain property, and it also reduces the design complexity by simply stacking the MTM superstrate layer on top of the patch antenna. In order to verify the effectiveness of the MTM-integrated rectenna, a prototype MTM-integrated rectenna is designed, fabricated, and characterized. The optimized near-zero property of the MTM unit cell is obtained using a parametrical analysis. In addition, the effects of the number of MTM unit cells on the performance of the integrated rectenna are studied. The conventional patch antenna is compared with the patch antenna integrated with the MTM superstrate to verify the focusing property of the MTM superstrate. Finally, the rectenna properties of the MTM-integrated rectenna are characterized and compared with those of the previously reported studies.

This paper is an extended work of the preliminary study [25]. Compared with [25], more results and discussions are reported, including the following: (1) the MTM-integrated rectenna is newly designed and fabricated on an RO5880 substrate to improve its rectenna performance, (2) parametric analysis is performed on the design of the MTM superstrate, (3) the studies on the effects of the number of MTM unit cells on the performance of the integrated rectenna is carried out, and (4) the detailed comparison of the MTM-integrated rectenna with previously reported rectenna systems is provided.

This paper is organized as follows. Section 2 discusses the design and analysis of the MTM integrated high-gain rectenna, and Section 3 presents the fabrication of the MTM-integrated rectenna. The simulated and measured results are presented in Section 4. Section 5 is the conclusion of this work.

## 2. Design and Analysis of the MTM Integrated High-Gain Rectenna

**Figure 2:**
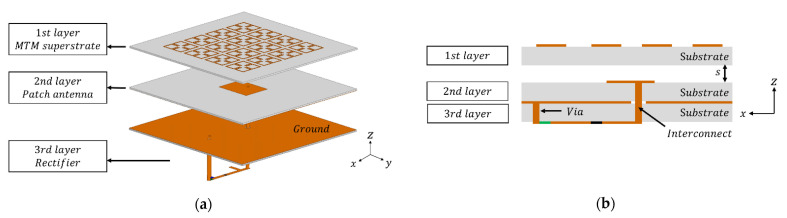
Schematic of a 3D integrated rectenna: (**a**) perspective view, (**b**) side view, where s=10 mm.

The schematic of the MTM integrated high-gain rectenna is presented in Figure 2. As shown in Figure 2a, the MTM-integrated rectenna consists of 1st layer of the MTM superstrate, 2nd layer of the patch antenna, and 3rd layer of the rectifier circuit. The MTM superstrate is stacked on the patch antenna with the separation s. The rectifier circuit is connected to the coaxial feeding point of the patch antenna through the interconnect.

### 2.1. Design of the MTM Unit Cell

In this subsection, the MTM unit cell is designed to have the gain improvement property. In this work, a 4-leaf clover shaped MTM unit cell [21] is carefully designed to have a near-zero refractive index for 2.45 GHz, a popular industry, science, and medicine (ISM) band, where multiple applications such as Wi-Fi, Bluetooth, and ZigBee are used and its RF waves are abundant around us, and the unit cell is designed on an RO5880 substrate (ε = 2.2, δ = 0.0009) instead of FR4 (ε = 4.4, δ = 0.03) in order to minimize the substrate loss. To design the MTM unit cell that has the near-zero refractive index at 2.45 GHz, a parametric analysis is performed using High Frequency Structure Simulator (HFSS, Ansys Inc. Canonsburg, PA, USA). In the parametric analysis, the effective refractive index can be obtained from the simulation results by using the standard retrieval methods [26,27,28].

(1) $$z=\pm \sqrt{\frac{{\left(1+S_{11}\right)}^{2}-S_{21}{}^{2}}{{\left(1-S_{11}\right)}^{2}-S_{21}{}^{2}}}\;\;\;$$

(2) $$e^{ink_{0}d}=\frac{S_{21}}{1-S_{11}\frac{z-1}{z+1}}$$

(3) $$n_{eff}=\frac{1}{k_{0}d}\{\left[{\left[\ln\left(e^{ink_{0}d}\right)\right]}^{\prime \prime }+2m\pi \right]-i{\left[\ln\left(e^{ink_{0}d}\right)\right]}^{\prime }\}$$

where *S*_11_ and *S*_21_ are the reflection and transmission coefficients; ${\left(\cdot \right)}^{\prime }$ and ${\left(\cdot \right)}^{\prime \prime }$ denote the real part and imaginary part of the complex numbers, respectively; $n_{eff}$ is the effective refractive index;$\;k_{0}$ is the wavenumber; d is the maximum thickness of the slab; z is the impedance; and m is the integer related to the branch index of $n^{\prime }$.

**Figure 3:**
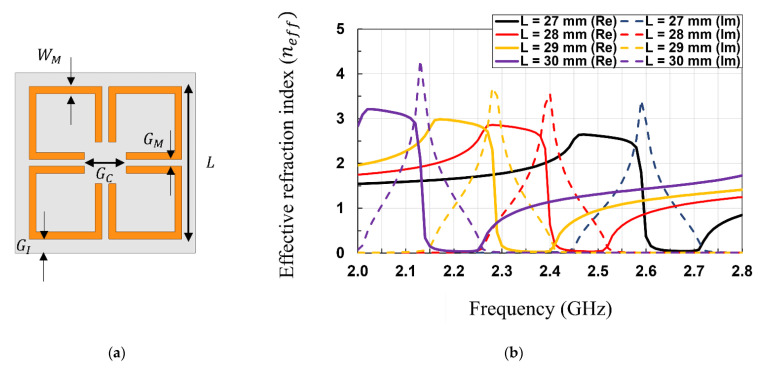
(**a**) Schematic of the 4-leaf clover shaped MTM unit cell; (**b**) Simulated effective refraction index of the MTM unit cell with the varied L values ($L=28\;\mathrm{mm},\;W_{M}=1\;\mathrm{mm},\;G_{M}=1\;\mathrm{mm},\;G_{C}=6\;\mathrm{mm},\;G_{I}=2\;\mathrm{mm}$ ).

Figure 3a shows the schematic of the 4-leaf clover shaped MTM unit cell, and Figure 3b shows the simulated effective refraction index of the MTM unit cell with the varied L values while keeping other variables same for simplicity. In this analysis, the near-zero range is defined as the range where the real values of the effective refractive index are equal to or less than 0.5 ($\eta_{eff}\left(re\right)\leq 0.5$) [25]. As shown in Figure 3b, the value of L is varied from 27 to 30 mm. When L increases, the near-zero range shifts to low frequencies, as depicted in Figure 3b. The L value is determined to be 28 mm, since the near-zero range of the MTM unit cell should be designed to include 2.45 GHz. As a result, the optimized near-zero range is shown to be from 2.4 to 2.54 GHz, which means the designed MTM changes the direction of the EM field by the boundary conditions to near-zero, thereby improving the gain of the antenna at the frequencies within this range.

### 2.2. Design of the MTM Integrated Patch Antenna

In this subsection, the MTM integrated patch antenna is designed and simulated to verify the gain improvement property of the MTM superstrate. As shown in Figure 1, the MTM superstrate is stacked on the patch antenna. Firstly, the 2nd layer of the patch antenna is designed to be 47.8 mm × 39.9 mm for 2.45 GHz. Then, the MTM superstrate is placed over the patch antenna with a separation of 10 mm. In order to optimize the number of the MTM unit cells, the effects of the number of MTM unit cells on the performance of the integrated rectenna are investigated. We compare the radiation patterns of the patch antenna with a 2 × 2, 3 × 3, 4 × 4, and 5 × 5 MTM arrayed superstrate and without an MTM superstrate.

**Figure 4:**
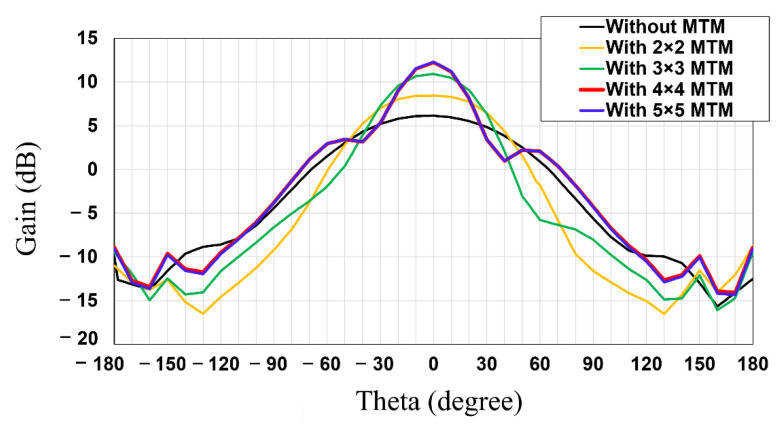
Simulated radiation patterns of the patch antenna with and without an MTM superstrate.

As shown in Figure 4, the gain of the patch antenna with a 4 × 4 MTM superstrate shows the highest peak gain, followed by the peak gain of the patch with a 5 × 5 MTM superstrate, and that with a 3 × 3 MTM superstrate, and that with a 2 × 2 MTM superstrate, and that without an MTM superstrate. The simulated peak gains of the patch antenna without and with an MTM superstrate are summarized in Table 1. As the number of the MTM unit cells increases, the peak gain tends to increase except for the case of the patch with the 5 × 5 MTM superstrate. Interestingly, the peak gain of the patch with the 5 × 5 MTM superstrate is slightly inferior to that with the 4 × 4 MTM superstrate. When the number of MTM unit cells in the array increases to 5 × 5, we expected the EM beam focusing coverage to be extended. However, since the beam focusing coverage of the 4 × 4 MTM superstrate is already enough to cover the size of the patch antenna, the peak gain improvement with the 5 × 5 MTM superstrate could be minute. Moreover, the simulation result indicates that the 5 × 5 MTM superstrate has increased loss compared with the 4 × 4 MTM superstrate, thereby decreasing the resultant peak gain of the antenna. Based on this analysis, it is concluded that the optimized number of the MTM unit cell in the array for the MTM superstrate is 4 × 4. Furthermore, it is worth noting that the number of MTM units could be determined depending on applications.

### 2.3. Design of the Rectifier Circuit

**Figure 5:**
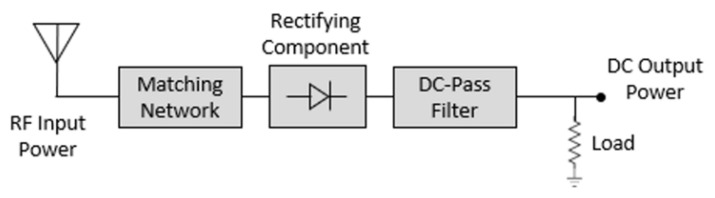
Block diagram of the rectifier circuit.

Figure 5 shows the block diagram of the rectifier circuit. A designed half-wave rectifier includes the matching circuit, rectifying component, DC-pass filter, and load. In this work, the rectifier is designed to have maximum RF–DC conversion efficiency at an input power of 10 dBm in order to provide sufficient output power to the end IoT devices. For the performance of the rectifier circuit, we utilize an HSMS2860 Schottky diode, which has a maximum forward voltage of 0.35 V. The rectifier circuit also contains a low-pass filter (100 pF shunt capacitor) and a resistive load (1.1k ohms). The choice of the capacitor and resistor values needs to fulfil a number of requirements. Firstly, the values must be chosen considering its cut-off frequency as its time constant should be very much longer than the time interval between the successive peaks of the rectified waveform. The value of the capacitor does not significantly affect the RF–DC conversion efficiency as long as the aforementioned criteria is satisfied. However, as for the value of the resistor, the optimum load resistor varies with frequency and input power. Here, the value of the load resistor and capacitor are optimized in Advanced Design System (ADS) software under an input power of 10 dBm to maximize the RF–DC conversion efficiency at 2.45 GHz. By sweeping the load resistance from 0.5 to 5 k ohms and the capacitance from 50 to 200 pF during the circuit optimization, the optimal values are found to be 1.1 k ohms for a resistor and 100 pF for a capacitor so that the conversion efficiency is the highest. The calculated cut-off frequency of the low-pass filter using selected values of the capacitor and resistor becomes 1.45 MHz, which can block high frequency band effectively. In addition, in order to ensure the maximal power transfer to the diode, a matching circuit is carefully designed at the diode input port. The impedance matching circuit is a crucial and also difficult part of the rectifier design. Due to the nonlinearity of the rectifier, the input impedance of the rectifier varies with the frequency, input power level, etc. In this work, an open stub is utilized to match the input impedance at 2.45 GHz at an input power of 10 dBm. The rectifier circuit is matched to 50 ohms since the input impedance of the MTM integrated antenna is 50 ohms.

**Figure 6:**
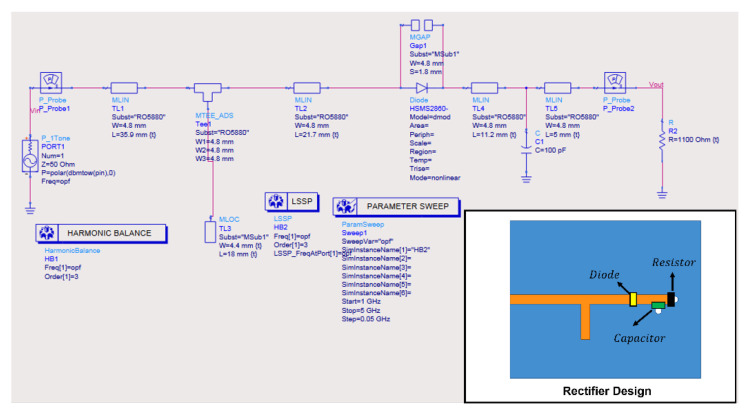
ADS schematic of the rectifier circuit.

As shown in Figure 6, ADS has been utilized for the simulation of the rectifier circuit. In order to consider the nonlinearity of the Schottky diode, Harmonic Balance simulation was utilized. In addition, the HSMS2860 spice model was utilized to obtain accurate simulation results. The detailed simulation results of the rectifier circuit are further discussed with the measurement results in Section 4.

## 3. Fabrication of the MTM Integrated High-Gain Rectenna

**Figure 7:**
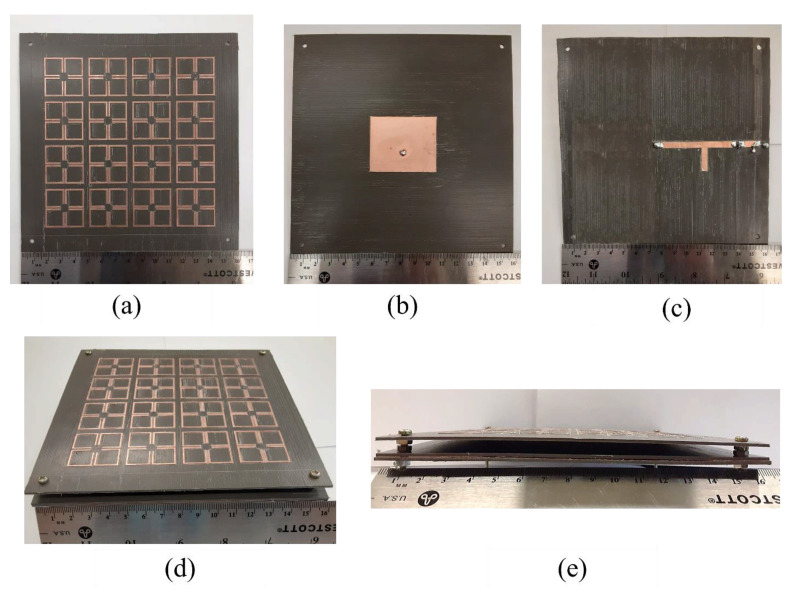
(**a**) Fabricated MTM superstrate, (**b**) fabricated patch antenna, (**c**) fabricated rectifier, (**d**) fabricated MTM-integrated rectenna, (**e**) side view of the fabricated MTM-integrated rectenna.

The prototype of the MTM integrated high-gain rectenna is fabricated on an RO5880 substrate. Each layer of the MTM-integrated rectenna is fabricated using a milling machine. Figure 7a–c shows the fabricated MTM superstrate, patch antenna, and rectifier circuit. Then, the patch antenna is fully integrated with the MTM superstrate and rectifier circuit, as shown in Figure 7d,e. For the fabrication of the rectifier circuit, The Schottky diode (HSMS 2860, Avago Technologies Inc. San Jose, CA, USA), 1.1k ohms resistor (ERA-6AEB112V, Panasonic Electronics Inc. Kadoma, Osaka, Japan), and 100 pF capacitor (GCM1885G2A101FA16D, Murata Electronics Inc. Nagokakyo, Kyoto, Japan) are selected.

## 4. Simulated and Measured Results

In this section, we evaluate the performances of the MTM integrated high-gain rectenna and compare the measured results with the simulation results.

### 4.1. Simulated and Measured Results of the Patch Antenna with and without MTM Superstrate

**Figure 8:**
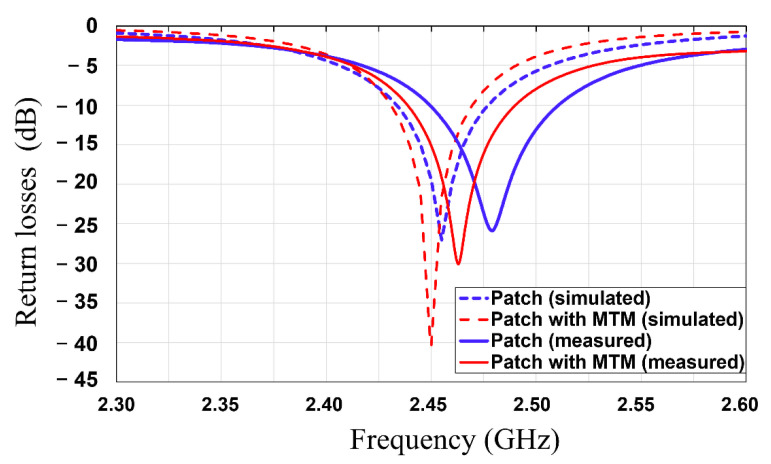
Simulated and measured return loss results of the patch antenna without and with MTM superstrate.

**Figure 9:**
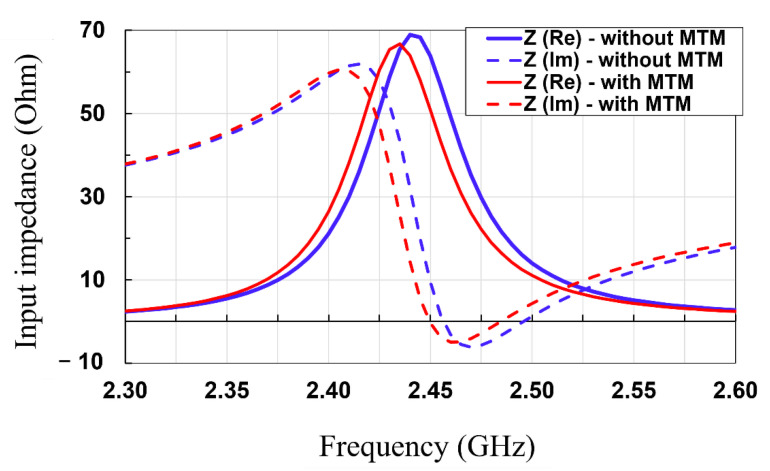
Simulated input impedance of the patch antenna without and with MTM superstrate.

In this subsection, the patch antenna with and without an MTM superstrate are simulated and measured, and their characteristics are compared. The return loss and peak gain of the antennas were measured using a vector network analyzer (HP E8361A, Agilent, Inc. Santa Clara, CA, USA) after standard one port short-open-load (SOL) calibration between 2.3 GHz and 2.6 GHz. Figure 8 shows the measured and simulated return losses of the patch with and without a 4 × 4 MTM superstrate. It is shown that the measured results are matched well with the simulated results. The measured resonant frequencies are a bit upshifted compared with the simulated ones, which are attributed to fabrication tolerance, i.e., the dimension of the fabricated devices is decreased during milling machine fabrication. In addition, the input impedances of the patch antenna with and without the MTM superstrate are simulated, as shown in the Figure 9. It is shown that, without MTM superstrate, the input resistance lies in the vicinity of 50 ohms with input reactance approximately near-zero at 2.46 GHz. With the MTM superstrate, the input resistance is shifted closer to ideal value of 50 ohms, and input reactance crossed zero at 2.45 GHz. Thus, the resonant frequency of the patch with the MTM superstrate is a bit downshifted compared with the one of the patch without MTM superstrate.

**Figure 10:**
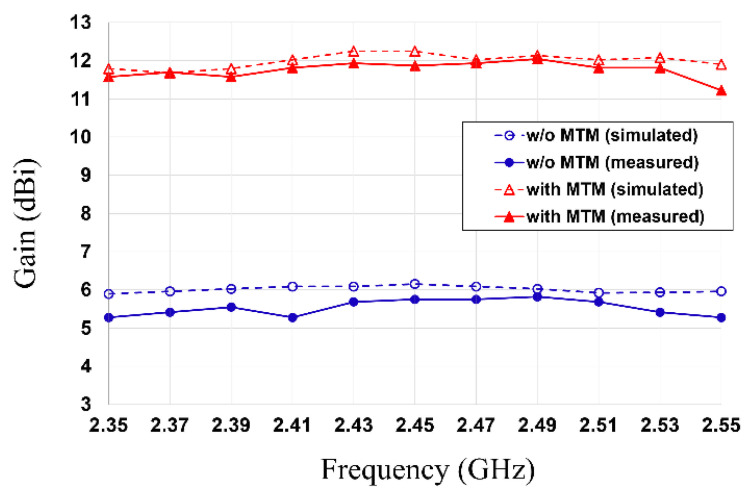
Simulated and measured peak gain of the patch antenna with and without MTM superstrate.

In addition, Figure 10 exhibits the simulated and measured peak gain of the antenna without and with an MTM superstrate as a function of frequency. The measured peak gain of the patch antenna is greatly enhanced from 5.75 dBi to 11.87 dBi (6.12 dB improvement) when the MTM superstrate is integrated on top of the patch. It means that the patch antenna architecture with an MTM superstrate can transfer 4.09 times as much power as the patch antenna without an MTM superstrate. The simulated and measured results in this section prove that the implemented MTM superstrate can serve as the beam-focusing lens, and the MTM superstrate is very effective for enhancing the antenna gain. The measured and simulated return loss and peak gain results are summarized in Table 2.

### 4.2. Simulated and Measured Results of the Rectifier Circuit

**Figure 11:**
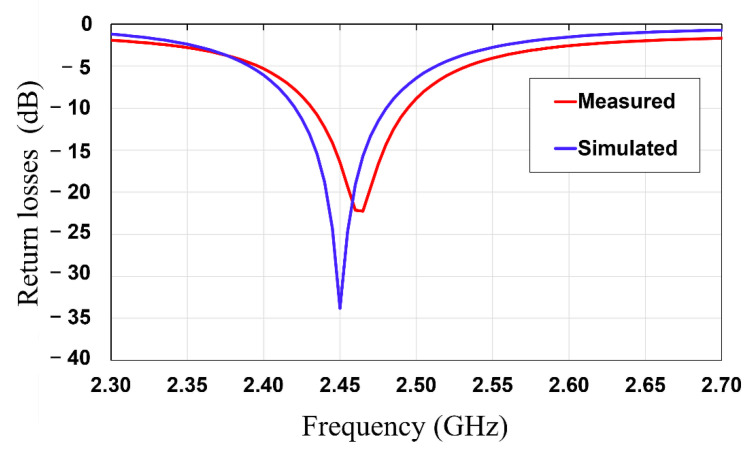
Simulated and measured return loss results of the rectifier circuit.

In this subsection, the third layer of the rectifier circuit is simulated, measured, and characterized. The ADS software was utilized for the simulation of the rectifier circuit. The measurement was performed using a vector network analyzer (HP E8361A, Agilent, Inc. Santa Clara, CA, USA), RF signal generator (HP 8648D, Agilent Inc. Santa Clara, CA, USA), and multimeter (Fluke 189). Firstly, the return loss of the rectifier circuit was simulated and measured using a vector network analyzer. Figure 11 shows that the measured results of the rectifier circuit exhibit a return loss of 22.4 dB at 2.47 GHz, which matches well with the simulated return loss of 33.6 dB at 2.45 GHz, which means the matching network of the rectifier matches well with the antenna.

**Figure 12:**
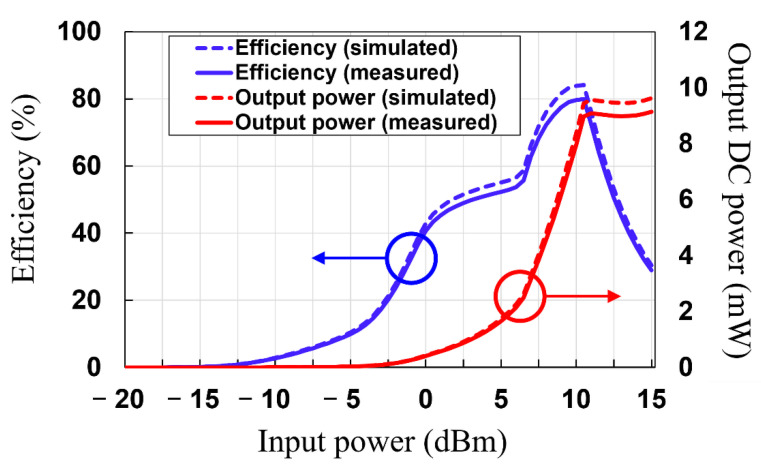
Simulated and measured return loss results of the rectifier circuit.

Then, the RF–DC conversion efficiency of the rectifier circuit was simulated and measured. The RF–DC conversion efficiency of the rectifier can be obtained using the following Equation (4):

(4) $$\eta_{RF-\mathrm{dc}}=\frac{P_{out}}{P_{in}}\times 100\;\left(\%\right)=\frac{V_{DC}{}^{2}}{P_{in}R_{L}}\times 100\;\left(\%\right),$$

where $P_{out}$ is the output DC power, $P_{in}$ is the input RF power to the rectifier circuit, $R_{L}$ is the load resistance value, and $V_{DC}$ is the output DC voltage. The simulated and measured RF–DC conversion efficiency and output DC power as a function of the input RF power levels to the rectifier circuit are depicted in Figure 12. The measured RF–DC conversion efficiencies are shown to be 2.69%, 40.71%, and 79.7% at −10 dBm, 0 dBm, and 10 dBm, respectively. A maximum RF–DC conversion efficiency of 79.96% is obtained at an input power of 10.5 dBm. The differences between simulated and measured results in Figure 10 can be attributed to: (1) The insertion loss of the SMA connector, which is connected between the RF signal generator and the rectifier circuit. This loss has not been taken into account in the ADS simulation. (2) The tolerance of the spice simulation model provided by the vendor of HSMS 2860 Schottky diode.

### 4.3. Measured Results of the MTM Integrated High-Gain Rectenna

**Figure 13:**
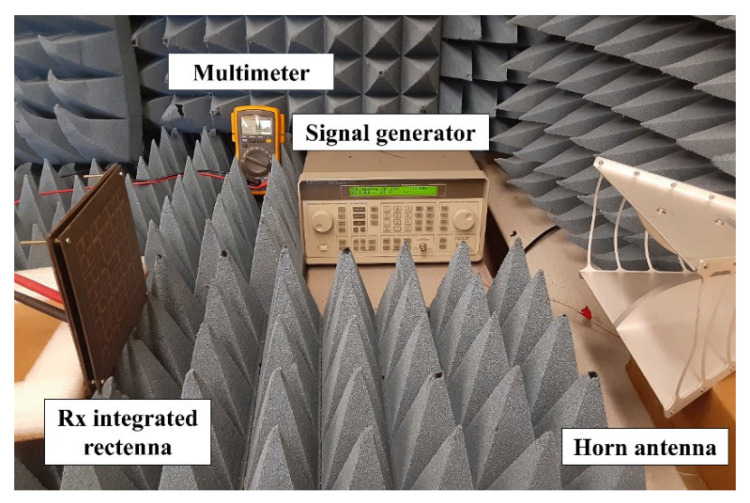
Measurement setup for the MTM-integrated rectenna.

Finally, the MTM integrated high-gain rectenna is fully integrated. Figure 13 shows the measurement setup of the MTM-integrated rectenna which consists of the RF signal generator, Tx horn antenna (JXTXLB-10180, A-INFO Inc. Irvine, CA, USA), Rx MTM-integrated rectenna, and multimeter. In the measurement, the MTM-integrated rectenna is placed at a distance of 40 cm away from the Tx antenna which satisfies the far-field condition at 2.45 GHz ($R\geq \frac{2D^{2}}{\lambda }=26.8\;\mathrm{cm}$, where D is the largest dimension of the aperture of the antenna and λ is the free-space wavelength at the operating frequency). The RF–DC conversion efficiency of the rectenna can be calculated by:

(5) $$\eta_{rectenna}=\frac{V_{L}{}^{2}}{P_{r}R_{L}}\times 100\;\left(\%\right)$$

where $P_{r}$ is the RF power captured by the Rx integrated rectenna, $R_{L}$ is load resistance value, and $V_{L}$ is output voltage on the load. The RF power captured by the Rx integrated rectenna, $P_{r}$, can be calculated by the Friis transmission equation:

(6) $$P_{r}={\left(\frac{\lambda }{4\pi r}\right)}^{2}G_{t}G_{r}P_{t}$$

where λ is the free-space wavelength at the operating frequency, r is the distance between Tx and Rx rectenna, $G_{t}$ and $G_{r}$ is the gain of the Tx and Rx antenna, respectively, and $P_{t}$ is the Tx power. In the measurement, the values of $G_{t}$ and $G_{r}$ are 11 dB and 11.87 dB, respectively.

**Figure 14:**
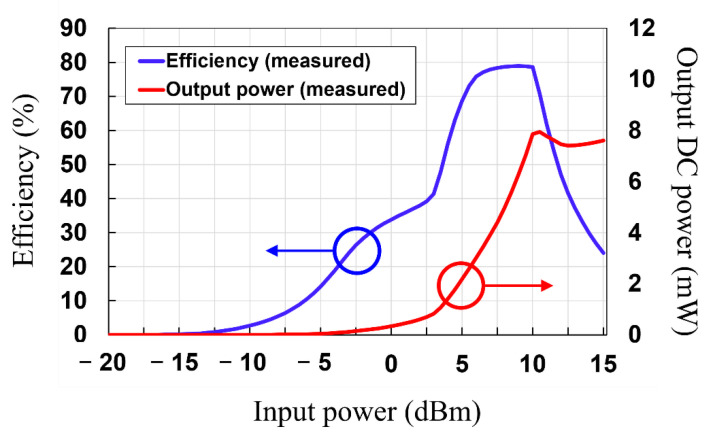
Measured RF–DC conversion efficiency of the MTM-integrated rectenna.

**Figure 15:**
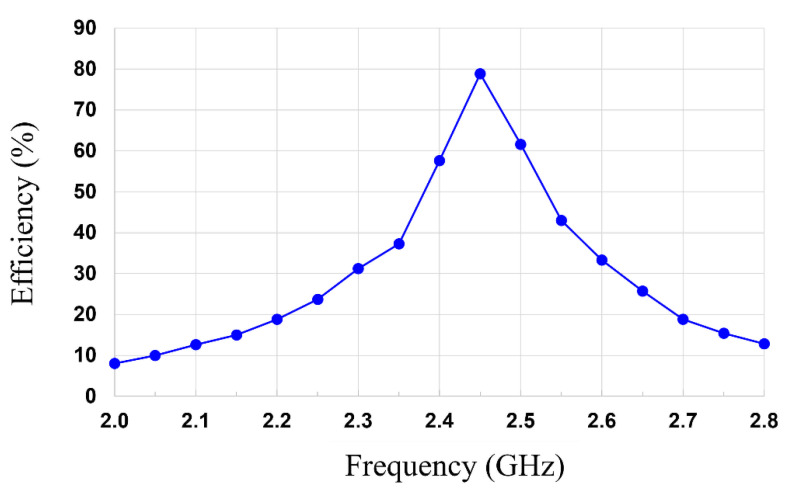
Measured RF–DC conversion efficiency versus frequency of the MTM-integrated rectenna.

As shown in Figure 14, the measured RF–DC conversion efficiency and output DC power of the MTM-integrated rectenna as a function of the RF input power can be achieved using the aforementioned analysis. It is shown that the measured RF–DC conversion efficiency of 2.67%, 33.84%, and 78.63 is obtained at −10 dBm, 0 dBm, and 10 dBm, respectively, with the maximum RF–DC conversion efficiency of 78.9% being at an input power of 9 dBm. When the input power of the rectenna without MTM is 2.88 dBm, the input power can be increased to 9 dBm by integrating MTM. This results in the improvement of the RF–DC efficiency from 39.2% to 78.9% and the increase of the output DC power from 0.7 mW to 6.27 mW (a factor of 8.96 improvements). Moreover, the RF–DC conversion efficiency versus the frequency at an input power of 10 dBm is plotted in Figure 15. It is shown that the measured RF–DC conversion efficiency is higher than 50% in the range of 2.38 to 2.53 GHz.

### 4.4. Comparison

In this subsection, the MTM integrated high-gain rectenna is compared with previously reported high-gain rectennas. Firstly, the demonstrated MTM-integrated rectenna is compared with the preliminary study [25] in Table 3. The MTM-integrated rectenna shows overall improved performance in terms of peak gain, improved gain, and RF–DC conversion efficiency, which are attributed to the decreased substrate loss. The peak gains without/with MTM are improved by 0.97 dB and 1.17 dB, respectively. This enhancement can be interpreted as the substrate loss difference between FR4 and RO 5880. Furthermore, the demonstrated MTM-integrated rectenna shows improved RF–DC conversion efficiency compared with [25], which is also due to the reduced substrate loss of RO5880.

The demonstrated MTM-integrated rectenna is compared with the previously reported high-gain rectennas. Comparison parameters include the operating frequency; a technique that is used for the rectenna design; diode; antenna dimensions; RF input power/density (P_in_/S); RF–DC conversion efficiency; design complexity/rectenna integration; and antenna gain. As shown in Table 4, the demonstrated MTM-integrated rectenna has shown outstanding performance compared to previously reported work. It is especially worth emphasizing that this is the first rectenna system utilizing the MTM superstrate integrated with the patch antenna and rectifier in a single device. Furthermore, the demonstrated work shows comparably high antenna gain and RF–DC conversion efficiency among selected work. Although the rectenna system demonstrated in [17] shows the highest gain, the rectenna exhibits a low RF–DC conversion efficiency of 42%, which leads to low end-to-end efficiency. In addition, the demonstrated MTM-integrated rectenna has a low design complexity compared with other work, as the MTM superstrate layer is integrated on top of the simple patch antenna and rectifier circuit.

## 5. Conclusions

In this work, the MTM integrated high-gain rectenna is demonstrated. The novel MTM superstrate approach with a three-layered integration method is firstly introduced for rectenna applications. The optimized near-zero property of the MTM unit cell is obtained using a parametric analysis. In addition, the effects of the number of MTM unit cells on the performance of the integrated rectenna are studied. By integrating the MTM on top of the patch antenna, the gain of the antenna is enhanced by 6.12 dB, resulting in the increase of incident RF power at the rectifier input, thereby inducing high-output DC power and high end-to-end efficiency. A measured RF–DC conversion efficiency of 2.67%, 33.84%, and 78.63 is achieved at −10 dBm, 0 dBm, and 10 dBm, respectively. A maximum RF–DC conversion efficiency of 78.9% is obtained at an input power of 9 dBm. When the input power of the rectenna without MTM is 2.88 dBm, the input power can be increased to 9 dBm by integrating MTM. This results in the improvement of the RF–DC efficiency from 39.2% to 78.9% and the increase of the output DC power from 0.7 mW to 6.27 mW (a factor of 8.96 improvements). The demonstrated MTM-integrated rectenna has shown outstanding performance compared to previously reported other work. It is especially worth emphasizing that this is the first rectenna system utilizing the MTM superstrate integrated with the patch antenna and rectifier in a single device. The demonstrated MTM-integrated rectenna has a low design complexity compared with other work, as the MTM superstrate layer is integrated on top of the simple patch antenna and rectifier circuit. In addition, the number of MTM units can be determined depending on applications. It is highly envisioned that the demonstrated MTM-integrated rectenna will provide new possibilities for practical energy harvesting applications with improved antenna gain and efficiency in various IoT environments.

## Figures and Tables

**Table 1:** Simulated peak gains of the patch antenna without and with an MTM superstrate.

Case	Peak Gain (dBi)	Improved Peak Gain (dB)
Patch only	6.15	-
Patch with 2 × 2 MTM superstrate	8.44	2.29
Patch with 3 × 3 MTM superstrate	10.93	4.78
Patch with 4 × 4 MTM superstrate	12.3	6.15
Patch with 5 × 5 MTM superstrate	12.25	6.1

**Table 2:** Simulated and measured performances of the patch antenna with and without MTM superstrate.

Parameters	Patch Only		Patch with MTM	
	Simulated	Measured	Simulated	Measured
Return loss (dB)	−27.05	−25.88	−40.34	−30.13
Resonant frequency (GHz)	2.46	2.479	2.45	2.465
10-dB bandwidth (GHz)	0.039	0.044	0.051	0.061
Peak gain (dBi)	6.15	5.75	12.3	11.87

**Table 3:** Comparison of This Work with Preliminary Work.

Parameters	[25]	This Work
Substrate	FR4 (ε = 4.4, δ = 0.03)	RO5880 (ε = 2.2, δ = 0.0009)
Operating Frequency	2.45 GHz	2.45 GHz
Dimensions (mm^3^)	150 × 150 × 14.71 (0.18 λ^3^)	158 × 158 × 14.71 (0.199 λ^3^)
Peak gain without MTM	4.78 dBi	5.75 dBi
Peak gain with MTM	10.7 dBi	11.87 dBi
Improved gain	5.92 dB	6.12 dB
Resistive load	1 kΩ	1.1 kΩ
Peak RF–DC conversion efficiency	63.5% @ 10.5 dBm input	78.9% @ 9 dBm input
DC power at peak efficiency	6.35 mW	6.27 mW

**Table 4:** Comparison of This Work with Other High-Gain Rectennas.

Ref.	Operating Frequency (GHz)	Technique	Diode	Antenna Dimensions (mm^3^)	P_in_ (dBm)/ S (µW/cm^2^)	RF–DC Conversion Efficiency (%)	Design Complexity/Rectenna Integration	Antenna Gain (dBi)
[12]	5.8	Reflecting surface	MA40150-119	-	20/-	80	High/Yes	10.7
[4]	2.45	Reflecting surface	HSMS2852	110 × 90 × 20.6 * (0.113 λ^3^)	-/1.95	80.03	Low/Yes	8.6
[13]	0.9	Differential patch	-	137 × 137 × 21.2 (0.01 λ^3^)	2.19/-	65.3	High/No	8.5
[16]	0.95	Antenna array	HSMS285C	165 × 165 × 0.8 (0.18 λ^3^)	5/-	76	High/No	8.5
[17]	24	SIW cavity	MA4E1317	55 × 55 × 0.5 * (0.776 λ^3^)	12.55/-	42	High/Yes	12.6
[9]	2.4	Air gap, aperture coupling	HSMS286B	110 × 89 × 5.07 (0.026 λ^3^)	10/-	32.52	Intermediate/No	7.82
[18]	5.78	Aperture coupling	HSMS2860	40 × 40 × 1.6 (0.026 λ^3^)	13.98/-	63	High/No	7
This work	2.45	MTM superstrate	HSMS2560	158 × 158 × 14.71 * (0.199 λ^3^)	9/-	78.9	Low/Yes	11.87

* Includes the rectifier dimension as the rectenna integrated in a single device.

## Data Availability

Not applicable.
